# L‐shaped morphology is a key risk factor for delamination in degenerative full‐thickness rotator cuff tears

**DOI:** 10.1002/ksa.12640

**Published:** 2025-03-03

**Authors:** Ethem Burak Oklaz, Ulunay Kanatli, Furkan Aral, Ramazan Duzgun, Oguzhan Ak, Muhammed Sakir Calta, Ali Turgay Cavusoglu

**Affiliations:** ^1^ Department of Orthopaedics and Traumatology Gazi University Faculty of Medicine Ankara Turkey

**Keywords:** delamination, full‐thickness rotator cuff tears, L‐shaped, shoulder arthroscopy, tear pattern

## Abstract

**Purpose:**

To evaluate the relationship between delamination and tear patterns in degenerative full‐thickness rotator cuff tears.

**Methods:**

This retrospective cohort study was conducted on patients who underwent shoulder arthroscopy for rotator cuff tears between December 2020 and September 2024. The study included chronic, degenerative, full‐thickness rotator cuff tears without concomitant shoulder pathology. Patients were grouped based on the presence of delamination, defined as the horizontal cleavage of the torn tendons. Age, sex, dominant extremity, symptom duration, amount of retraction, tear width and tear pattern (crescent‐shaped, anterior L‐shaped, posterior L‐shaped and U‐shaped) were compared between groups. Regression analysis was conducted to identify risk factors that may be associated with the presence of delamination.

**Results:**

The study included 130 patients, 75 with delamination (mean age 61.1 ± 8.8 years) and 55 without (mean age 61.1 ± 8.3 years). Demographic characteristics were similar among patients with and without delamination. The rate of anterior and posterior L‐shaped tears was significantly higher in delaminated tears (24% and 33%, respectively) compared to non‐delaminated tears (6% and 9%, respectively) (*p* = 0.003 and *p* = 0.001, respectively). Regression analysis demonstrated that anterior L‐shaped tears and posterior L‐shaped tears were significantly related to delamination (*p* = 0.002 and *p* = 0.001, respectively).

**Conclusion:**

This study demonstrates that anterior and posterior L‐shaped tear patterns are significantly associated with delamination in degenerative full‐thickness rotator cuff tears.

**Level of Evidence:**

Level III, retrospective cohort study.

## Graphical abstract

Anterior and posterior L‐shaped tear patterns are significantly associated with delamination in degenerative full‐thickness rotator cuff tears. (a) Apex of the tear. (b) Most medial edge of the tear. (c) Origin of the tear.

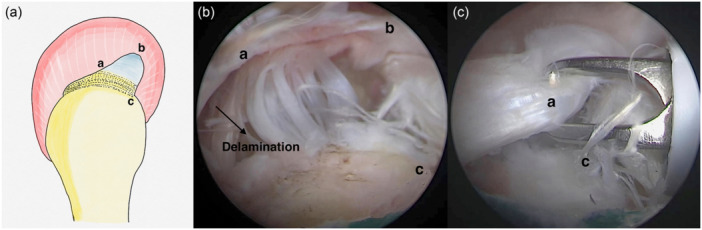

**Abbreviations**
AUC: area under the curve
OR: odds ratio
ROC: receiver operating characteristic

## INTRODUCTION

Since the recognition of the importance of arthroscopic lateral portal assessment in examining rotator cuff tears, the reported prevalence of delamination has increased significantly, ranging from 32% to 92% [2, 9, 13, 14, 16]. Delamination refers to a horizontal tear or cleft in the tendon, caused by incompatible tension forces acting on the bursal and articular surfaces [2, 25, 29]. The separation between surfaces results in the formation of a structurally distinct tear consisting of two layers [2, 12, 13]. Studies have indicated that these layers should be carefully managed during the arthroscopic repair process and may require surgical techniques different from those used for non‐delaminated tears [2, 7, 8, 10, 13, 21, 22]. These considerations make delamination a critical finding that surgeons repairing rotator cuff tears should be particularly aware of.

Although delamination was first described three decades ago, most studies have focused on tendon repair techniques, leaving the tear characteristics of delamination unexplored. Among these characteristics, the tear pattern (crescent‐shaped, anterior L‐shaped, posterior L‐shaped or U‐shaped) is one of the important features to consider when evaluating tear morphology. This is because each tear pattern represents a different configuration, necessitating tailored modifications to repair strategies [6, 15, 18, 27, 30, 31]. Furthermore, the literature indicates that anterior L‐shaped tears demonstrate significant tension differences between the articular and bursal layers, which may lead to the occurrence of delamination [24]. Given that both delamination and tear patterns affect the choice of surgical technique, identifying specific tear shapes associated with delamination could assist surgeons in determining which patients require special attention, enabling the most appropriate surgical approach to be employed.

Therefore, the aim of the present study was to investigate the relationship between tear patterns and delamination in full‐thickness rotator cuff tears. The hypothesis of this study was that L‐shaped tear patterns are significant risk factors for delamination.

## METHODS

Institutional review board approval was received from the Gazi University Ethics Committee (Protocol: 2024‐1393). The study adhered to the guidelines of the World Medical Association's Declaration of Helsinki.

**Figure 1:**
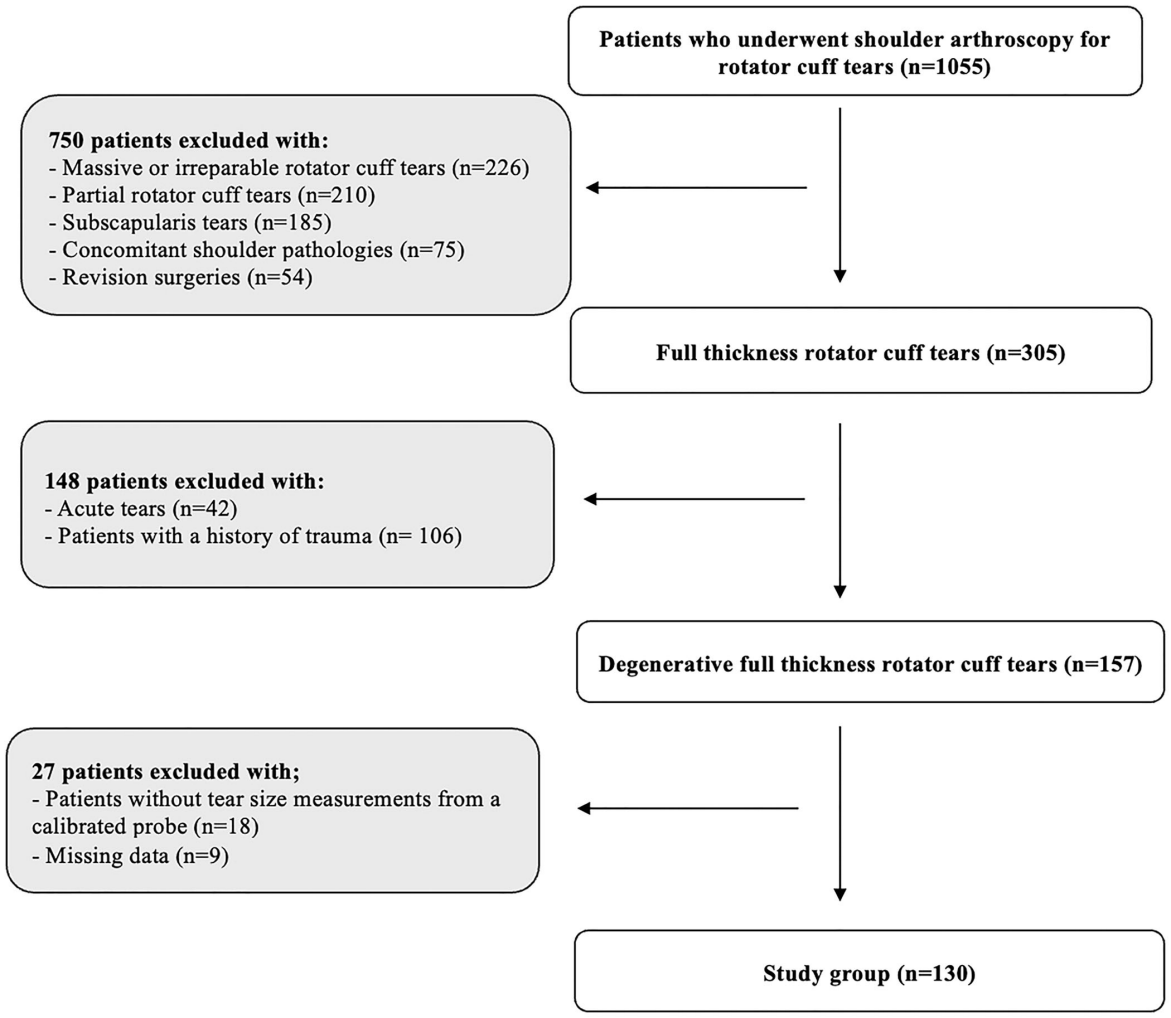
Flowchart of the study.

Patients who were admitted to the institution with a diagnosis of rotator cuff tears and underwent arthroscopic surgery by a single senior surgeon (U.K.) between December 2020 and September 2024 were retrospectively evaluated. The inclusion criteria were as follows: (1) patients with chronic full‐thickness rotator cuff tears, (2) patients without a history of trauma and (3) patients with adequate and accessible data, including preoperative x‐rays, examination findings (trauma history and symptom duration), and surgical video recordings. The exclusion criteria were as follows: (1) patients with massive or irreparable rotator cuff tears, (2) patients with partial rotator cuff tears, (3) patients with subscapularis tears, (4) patients with concomitant shoulder pathologies (e.g., labral tears, glenohumeral osteoarthritis or inflammatory arthritis), (5) patients with acute tears, (6) patients with a history of prior surgery on the same shoulder, (7) patients whose tear size was not measured intraoperatively with a calibrated probe and (8) patients with missing data (preoperative examination findings and surgical video recordings). The patient selection process is described in the flowchart (Figure 1).

**Figure 2:**
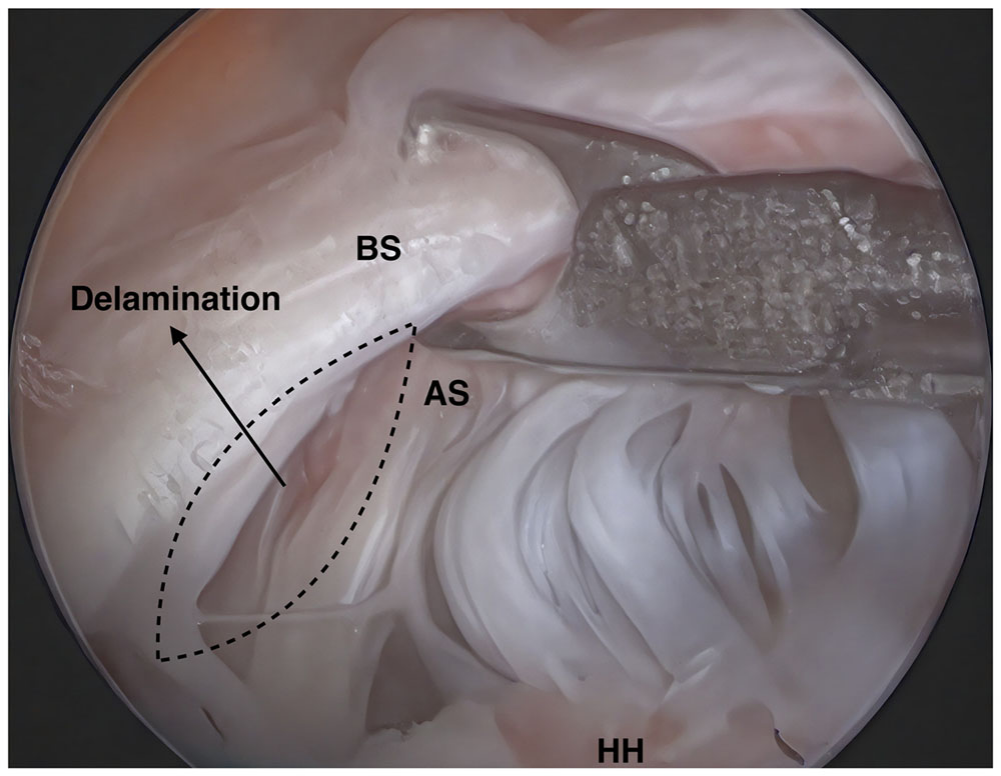
Arthroscopic view of the right shoulder, with the patient in the lateral decubitus position and the arthroscope in the lateral viewing portal. The image illustrates a rotator cuff tear with delamination. AS, articular side; BS, bursal side; HH, humeral head.

**Figure 3:**
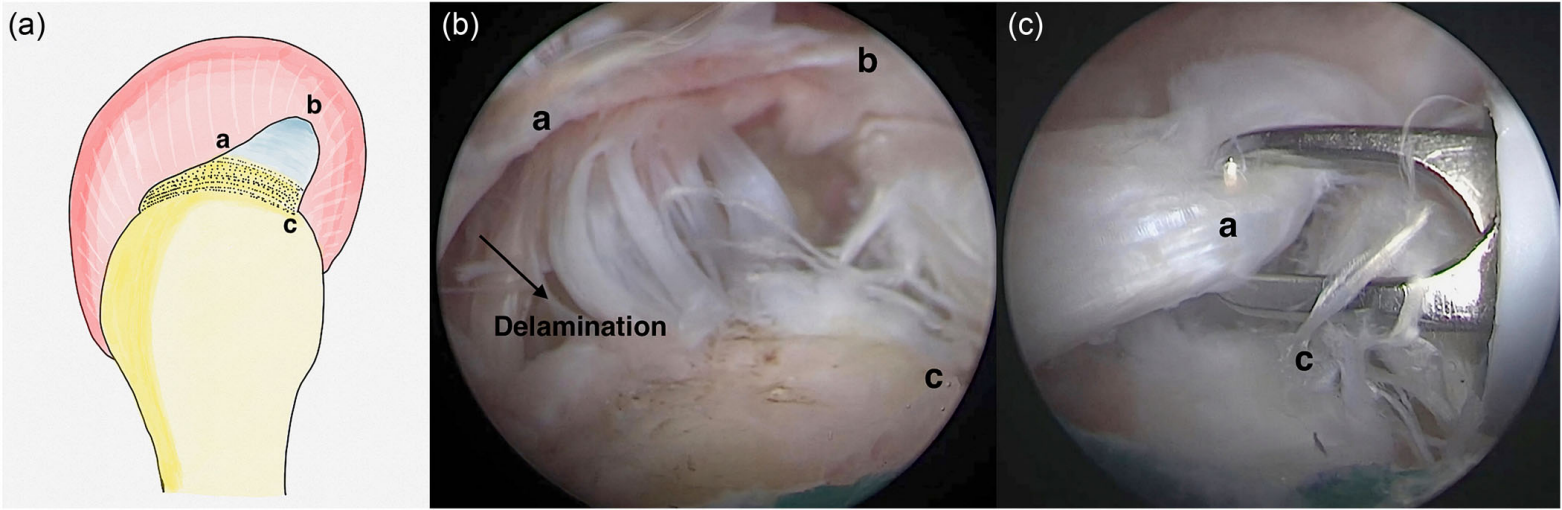
Arthroscopic view of the right shoulder, with the patient in the lateral decubitus position and the arthroscope in the lateral viewing portal. Image (a) illustrates a graphical representation of an anterior L‐shaped tear. Image (b) illustrates an arthroscopic view of an anterior L‐shaped tear. Image (c) shows that anatomical integrity has been restored by repositioning the apex of the anterior L‐type tear to its original position on the footprint.

Tears were defined as delaminated when edge fraying and cleavage tearing measured ≥5 mm (Figure 2) [17]. The tear patterns of the patients included in the study were determined by arthroscopic examination conducted by a senior surgeon (U.K.), and the tears were classified into four groups: (1) crescent‐shaped tears, (2) anterior L‐shaped tears (tears with a longitudinal component located anteriorly), (3) posterior L‐shaped tears (tears with a longitudinal component located posteriorly) and (4) U‐shaped tears [6, 18, 23]. The width of the tear (anteroposterior length) and the extent of medial retraction were measured using a calibrated probe during arthroscopic examination. In assessing retraction in crescent and U‐shaped tears, the most medial edge of the tear was identified as the apex, and retraction was defined as the distance from this point to the footprint on greater tuberosity [23]. In L‐shaped tears, the apex was defined as the midpoint of the tear edge, and retraction was measured as the distance from this apex to the point where the tear separated from the footprint on the greater tuberosity. (Figure 3) [23]. In patients with delamination, the width of the tear and the amount of retraction were measured separately for the bursal and articular sides. Retraction was determined by measuring the distance from the apex of the tear to the footprint on the greater tubercle for the bursal surface, and to a point 2 mm lateral to the articular cartilage for the articular surface [13]. The gap between the articular and bursal sides was measured as the maximum distance between the tear margins of the two layers [13].

### Statistical analysis

The relationship between delamination and variables, including age, sex, dominance, symptom duration, tear retraction, tear width and tear patterns, was analyzed. The Shapiro–Wilk test was used to assess the normality of the data. Characteristics were compared between groups using Pearson's chi‐square or Fisher's exact tests, depending on the data distribution. For the measurement of parameters including age, symptom duration, amount of retraction, tear width, and the gap between layers, the Mann–Whitney *U* test was used to compare values between groups in cases of non‐normal distribution, whereas the independent t‐test was applied for normally distributed data. Logistic regression analysis was performed to evaluate the risk factors for the presence of delamination. The receiver operating characteristic (ROC) curve and area under the curve (AUC) were used to evaluate the relationship between tear width and delamination for different tear patterns. During ROC analysis, tear sizes in the delamination group were calculated by averaging the bursal and articular side tears, and the mean width and retraction were determined for each patient. The degree of association was considered excellent for an AUC > 0.8, acceptable for an AUC > 0.7, poor for an AUC between 0.6 and 0.7 and failed for an AUC < 0.6 [1, 5]. All statistical analyses were performed using SPSS for Mac (Version 28.0; IBM Corp), and a *p* value < 0.05 was considered as statistically significant.

A chi‐square goodness‐of‐fit test for contingency tables was performed with an effect size (*w*) of 0.414, estimated based on preliminary observations within the pilot study. The analysis was conducted with a significance level (*α*) of 0.05 and a power (1 − *β*) of 0.80, yielding a required total sample size of 46. The noncentrality parameter (*λ*) was 7.88, and the critical chi‐square value was 3.84. The actual achieved power was 0.802, confirming that the sample size was adequate for detecting the hypothesized effect size within the pilot study.

## RESULTS

A total of 130 patients who met the inclusion criteria were included in the study. There were 75 delaminated and 55 non‐delaminated tears. No significant differences were observed in age, sex, surgery on the dominant extremity or preoperative symptom duration between patients with and without delamination (Table 1). Tear width and retraction were higher in patients with delamination (Table 1). However, tear retraction and width were similar for the bursal and articular sides of the delaminated tears (*p* = 0.9 and *p* = 0.06, respectively) (Table 1). In delaminated tears, the gap between the bursal and articular tear edges was 7.2 ± 4.8 mm. Majority of the tears (54%) were crescent‐shaped, while 23% were posterior L‐shaped, 16% were anterior L‐shaped and 7% were U‐shaped (Table 1). Tear patterns differed between groups, where the non‐delaminated group had a significantly higher proportion of crescent‐shaped tears. The difference was also apparent for the proportion of anterior and posterior L‐shaped tears in the delaminated group (Table 1).

**Table 1:** Characteristics of patients in the main group.

	Delamination		
Variable	Presence (*n* = 75)	None (*n* = 55)	*p* *
Age at surgery, yr (mean ± SD)	61.1 ± 8.8	61.1 ± 8.3	0.98
Sex, *n* (%) (male/female)	15 (20%)/60 (80%)	13 (24%)/42 (76%)	0.66
Dominance, *n* (%)			0.68
Dominant arm	58 (77%)	40 (73%)	
Non‐dominant arm	17 (23%)	15 (27%)	
Symptom duration, mo (mean ± SD)	32.5 ± 27.4	25.3 ± 23.6	0.10
Amount of retraction, mm (mean ± SD)		16.7 ± 8.6	**0.04**
Articular	19.9 ± 9.8		
Bursal	19.8 ± 8.8		
Tear width, mm (mean ± SD)		19.8 ± 8.2	**0.01**
Articular	24.0 ± 9.1		
Bursal	23.9 ± 9.1		
Tear pattern, *n* (%)			**<0.001**
Crescent‐shaped	28 (38%)	42 (76%)	**<0.001**
Anterior L‐shaped	18 (24%)	3 (6%)	**0.003**
Posterior L‐shaped	25 (33%)	5 (9%)	**0.001**
U‐shaped	4 (5%)	5 (9%)	0.31

*Note*: *p*‐values that were statistically significant are presented in bold.

Abbreviations: mm, millimetre; mo, month; SD, standard deviation; yr, year.

* Significant at *p* value ≤ 0.05.

Multivariate regression analysis demonstrated that anterior L‐shaped tears (odds ratio [OR] = 9.08; 95% confidence interval [CI] = 2.32–35.58; *p* = 0.002) and posterior L‐shaped tears (OR = 7.51; 95% CI = 2.41–23.39; *p* = 0.001) were significantly associated with delamination (Table 2). ROC analysis of the relationship between tear width and delamination across tear patterns indicated a significant correlation for anterior L‐shaped tears (AUC = 0.759; cut‐off = 19 mm; sensitivity = 83%; specificity = 67%). The AUC value for posterior L‐shaped tears (AUC = 0.652; cut‐off = 20.5 mm; sensitivity = 92%; specificity = 60%) demonstrated a weaker association than those for anterior tears. The AUC values for crescent‐shaped (AUC_Crescent shaped_ = 0.515) and U‐shaped (AUC_Ushaped_ = 0.225) tears were found to be failures.

**Table 2:** Logistic regression model assessing delamination based on patient characteristics in the main group.

Variable	Reference category	Analyzed category	Univariate			Multivariate		
			Odds ratio (Exp(*B*))	95% CI for Exp(B)	*p* *	Odds ratio (Exp(*B*))	95% CI for Exp(*B*)	*p* *
Age at surgery	–		1.000	0.960–1.042	0.98			
Sex	Male	Female	0.808	0.348–1.873	0.61			
Dominant extremity	No	Yes	1.279	0.573–2.855	0.54			
Symptom duration	–		1.012	0.997–1.027	0.12			
Amount of retraction	–		1.494	1.000–2.234	**0.05**	0.589	0.204–1.700	0.32
Tear width	–		1.662	1.102–2.506	**0.01**	1.939	0.682–5.514	0.21
Tear pattern	Crescent	Anterior L	9.000	2.422–33.438	**0.001**	9.085	2.320–35.580	**0.002**
		Posterior L	7.500	2.566–21.924	**<0.001**	7.514	2.413–23.398	**0.001**
		U	1.200	0.296–4.862	0.79			

*Note*: *p*‐values that were statistically significant are presented in bold.

Abbreviations: CI, confidence interval; Exp(*B*), exponentiated regression.

* Significant at *p* ≤ 0.05.

## DISCUSSION

The most important finding of this study is that L‐shaped morphology is significantly associated with delamination in degenerative full‐thickness rotator cuff tears. Therefore, in L‐shaped rotator cuff tears, delamination should be assessed through the lateral viewing portal to determine the most appropriate repair technique.

As is well known, multiple studies have demonstrated significant structural differences among various tear shapes and have emphasized that repair techniques should be planned on a tear‐specific basis [6, 8, 11, 20, 27]. However, in 2013, Sano et al. [24] provided a new perspective on the effect of tear shape on rotator cuff by investigating variations in stress distribution between different tear patterns. They analyzed the stress distribution in anterior L‐shaped and crescent‐shaped tears in a biomechanical study. As a remarkable finding, they reported that the equivalent stress differences between the articular and bursal layers of the tendon increased rapidly in anterior L‐shaped tears exceeding 2 cm in width. As differences in stress distribution between layers may lead to horizontal tears through shear stress, they stated that their findings could be associated with tendon delamination. The current study corroborates the findings of Sano et al.'s [24] biomechanical study by identifying a significant relationship between anterior L‐type tears wider than 19 mm and delamination, suggesting that rapid changes in stress distribution between layers may result in delamination. Another tear pattern associated with delamination has been identified as the posterior L‐shaped tears. Little is known in the literature about the effect of this tear type on stress distribution; however, two hypotheses could be proposed to explain its relationship with delamination: (1) common feature of anterior and posterior L‐shaped tears is their composition of two tear components: transverse and longitudinal. Although the position of the longitudinal component is different, these tears are mirror images of each other and have similar configurational characteristics [23]. Therefore, the stress distribution differences caused by posterior L‐shaped tears on the articular and bursal layers may be similar to those of anterior L‐shaped tears, potentially leading to delamination. (2) In the posterior region, which functions as a transition zone between the infraspinatus and supraspinatus tendons, structural differences between the tendons may result in variations in stress dynamics [3, 26]. Consequently, in this region, the presence of a longitudinal tear could significantly alter stress distribution, potentially leading to delamination.

The findings of the present study are important in enhancing orthopaedic surgeons' awareness of delamination when managing L‐shaped full‐thickness rotator cuff tears. This is because most studies have indicated that, in the repair of delaminated tears, particular techniques should be performed depending on the characteristics of the delamination [4, 7, 19, 21, 22]. In addition, the literature suggests that, for better intratendinous healing in delaminated tears, the synovial tissue within the horizontal cleft should be curetted [17, 28, 29]. The relationship between delamination and tear shape could also be looked at from the reverse perspective. When delamination is identified during arthroscopic examination, the potential presence of an L‐shaped tear should be thoroughly assessed. This is because the management of L‐type tears may require different repair techniques [6, 18, 20, 23]. Especially, repair of the longitudinal component using tendon‐tendon sutures is a specific and important part of the reconstruction. In summary, the combination of delamination and L‐shaped tears is relatively common, and the presence of one warrants an assessment of the other.

The critical point in these assessments is the necessity of examining both parameters through the lateral viewing portal. Han et al. [9] reported that delamination is often overlooked when tear examinations are performed solely through the posterior viewing portal and emphasized that evaluations using the lateral viewing portal are essential for the accurate detection of delamination. Similarly, Davidson and Burkhart [6] stated that the tear shape can be most reliably assessed through the lateral viewing portal. In line with the literature, data for all patients in the present study were obtained through evaluations conducted using the lateral viewing portal. To conclude, the morphology of full‐thickness rotator cuff tears should be carefully evaluated from the lateral viewing portal to accurately identify delamination and tear shape.

There are several limitations in this study. The retrospective design and reliance on data from a single surgeon's practice may introduce selection and information biases. Moreover, the proposed hypotheses about the relationship between posterior L‐shaped tears and delamination remain speculative. Further biomechanical studies investigating stress distribution in posterior L‐shaped tears are needed to determine the underlying cause of this relationship.

## CONCLUSIONS

This study demonstrates that anterior and posterior L‐shaped tear patterns are significantly associated with delamination in degenerative full‐thickness rotator cuff tears.

## AUTHOR CONTRIBUTION

Ulunay Kanatli, Ethem Burak Oklaz and Muhammed Sakir Calta conceptualized and designed the study. Furkan Aral, Ramazan Duzgun and Oguzhan Ak collected the data. Ethem Burak Oklaz and Ulunay Kanatli analyzed the data and drafted the manuscript. Ali Turgay Cavusoglu revised the manuscript. All authors reviewed, refined and approved the final manuscript.

## ETHICS STATEMENT

The study protocol was approved by the University Ethical Committee (Decision E‐77082166‐604.01‐1039865, research code: 2024‐1393).

## CONSENT

All participants consented to participate in the study.

## CONFLICT OF INTEREST STATEMENT

The authors declare no conflicts of interest.

## Data Availability

The data that support the findings of this study are available on request from the corresponding author (E.B.O.).
